# Can We Structure Biomaterials to Spray Well Whilst Maintaining Functionality?

**DOI:** 10.3390/bioengineering10010003

**Published:** 2022-12-20

**Authors:** Richard J. A. Moakes, Liam M. Grover, Thomas E. Robinson

**Affiliations:** Healthcare Technologies Institute, University of Birmingham, Birmingham B15 2TT, UK

**Keywords:** spray physics, structured fluid, colloids, controlled delivery, advanced materials

## Abstract

Structured fluid biomaterials, including gels, creams, emulsions and particle suspensions, are used extensively across many industries, including great interest within the medical field as controlled release vehicles to improve the therapeutic benefit of delivered drugs and cells. Colloidal forces within these materials create multiscale cohesive interactions, giving rise to intricate microstructures and physical properties, exemplified by increasingly complex mathematical descriptions. Yield stresses and viscoelasticity, typically arising through the material microstructure, vastly improve site-specific retention, and protect valuable therapeutics during application. One powerful application route is spraying, a convenient delivery method capable of applying a thin layer of material over geometrically uneven surfaces and hard-to-reach anatomical locations. The process of spraying is inherently disruptive, breaking a bulk fluid in successive steps into smaller elements, applying multiple forces over several length scales. Historically, spray research has focused on simple, inviscid solutions and dispersions, far from the complex microstructures and highly viscoelastic properties of concentrated colloidal biomaterials. The cohesive forces in colloidal biomaterials appear to conflict with the disruptive forces that occur during spraying. This review explores the physical bass and mathematical models of both the multifarious material properties engineered into structured fluid biomaterials and the disruptive forces imparted during the spray process, in order to elucidate the challenges and identify opportunities for rational design of sprayable, structured fluid biomaterials.

## 1. The Evolution of Biomaterials

The use of external materials to augment, repair, and replace parts of the body has been a part of society for millennia. The use of biomaterials in prehistoric humans is still debated, but they were certainly used in early civilisations. For example, the Etruscans produced the first dental bridges from simple gold bands as early as 630 BC [1]. These early biomaterials were used as inert replacements for lost body parts, such as glass eyes and iron hands; particularly famous examples include the ivory teeth of George Washington and the bronze nose of Tycho Brahe [2]. Medical advances in the 19th century, notably use of anaesthesia, aseptic technique and the X-ray, then paved the way for successful implantation of materials. Coupled with the boom in high-performance materials developed in the 20th century wars, new metal, ceramic and, perhaps most notably, polymeric materials were implanted as heart valves, joint replacements, stents, and more [3]. Over time, these inert solid implants, often existing materials from other industries repurposed by physicians for medical use, were replaced with biomaterials designed specifically for their given function, leading to modern biomaterials science and technology. The medical biomaterials field now covers innumerable applications, diseases, tissues and material types [4,5].

Today, colloidal systems are often utilised in biomaterials. These include solid particles, in the form of dispersions, suspensions and pastes, lipids, often utilised as emulsions, ointments and creams, and polymers, in the form of concentrated solutions, fluid gels and microgel suspensions. Notable properties of colloidal biomaterials, as discussed in Section 2, include the potential for shear-thinning, self-healing, viscoelasticity and stimuli-responsiveness. The chemical, structural and mechanical versatility of these materials make them the first choice for many biomedical applications.

Perhaps the most extensive use of colloidal biomaterials is in the controlled delivery of therapeutic drugs and cells. Controlled delivery allows a high, even therapeutic concentration at the target site but a low concentration elsewhere, limiting off-site side effects and improving therapeutic efficiency. Key roles of the biomaterial here include protecting valuable cargo from destructive chemical environments and mechanical forces during application, increasing both retention time and site specificity at the target location and, in the case of drugs, modifying release profiles. Drugs can be loaded directly into the material, in either the dispersed or continuous phase, or bonded to the particles or polymers, either ionically or covalently, for triggered release. Encapsulating the drug into an additional phase, such as a micro- or nanoparticle, can be used to decouple the material properties of the bulk biomaterial and the release profile of the therapeutic [6]. Drug release can then proceed through multiple mechanisms, including diffusion, swelling, and chemical cleavage [7].

Colloidal delivery systems, particularly those utilising hydrophilic polymers, have been employed for a vast range of biomedical applications. These include, but are by no means limited to, topical delivery to the eye [8,9] and skin [10,11], as well as rectal [12] and vaginal [13,14] routes of delivery. These materials have also been designed to be used internally for applications in oncology [15,16,17], wound healing [18], osteoarthritis [19] and bone repair [20,21].

**Figure 1:**
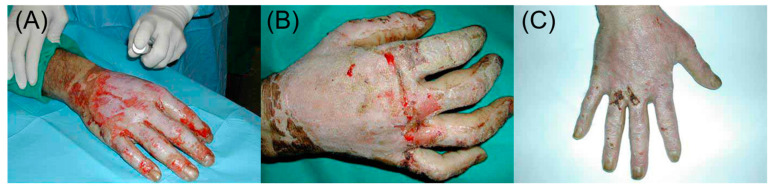
Example of a cell-spray-based regeneration of a deep partial thickness burn wound, showing (**A**) spray application of the cell system to the wound, and the results after (**B**) one week and (**C**) one month. Adapted from [28], reproduced with permission from Elsevier.

Spraying is an effective delivery method for many biomaterials. Particular advantages include high patient compliance and potential for self-administration, and the ability to apply a fine layer of material to uneven or hard-to-reach surfaces, particularly the internal mucosa. This layer can create a physical barrier to prevent bacterial and viral infection [22,23,24,25], as well as efficiently deliver high value therapeutics to a surface. Specific biomedical applications include spraying cells onto chronic and burn wounds for accelerated regeneration [26,27,28] (Figure 1), film forming sprays for topical drug delivery [25,29], buccal absorption for enhanced vitamin D3 uptake [30], sprays to prevent mucositis in the mouth [31] and infection in the vagina [32], as well as nasal sprays for local, systemic and central nervous system delivery [23,24,33].

Both increased structuring and spray delivery are becoming more popular in fluidic biomaterials, particularly in the field of controlled delivery. However, as the level of cohesive structure increases in modern biomaterials, the ability to readily disrupt them into sprays may diminish. This review therefore examines the forces and microstructures being increasingly employed in modern biomaterials, and the contemporary perspectives on spraying more complex fluids. By considering both fields simultaneously, with an emphasis on their mathematical descriptions, the challenges of spraying increasingly structured fluids, and the opportunities it could afford to the biomedical community, are discussed.

## 2. The Cohesive Forces in Structured Fluid Biomaterials

Simple, inviscid, Newtonian fluids have long been used in medicine. In the specific context of spraying, this includes the use of saline as a nasal irrigant [34], and delivery of drugs in aqueous solutions, such as eye mists [35] and pulmonary nebulisers [36]. However, these simple materials lack the functional benefits of structured fluids, which are being increasingly utilised in modern biomaterials for controlled delivery [27]. As discussed in Section 1, colloidal biomaterials are useful in the controlled delivery of drugs and cells, primarily due to their ability to create structure in the delivery medium. This can increase the specificity of delivery, providing higher amounts to the target site while reducing off-site effects, increase retention at the target site, prolonging the therapeutic effect, and protect drugs and cells from mechanical forces and environmental factors during delivery. Further, creating a colloidal network can alter drug release profiles and mimic the physiological environment for cells.

**Figure 2:**
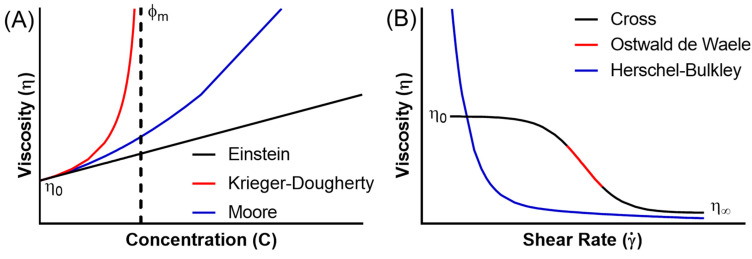
Graphs showing the change in viscosity with (**A**) concentration and (**B**) shear rate, described by common literature models.

At low concentrations, each entity, be it a solid particle, droplet or polymer, is discrete and sufficiently far apart from others such that the colloids do not interact with each other, and disrupt the flow of the continuous phase independently [37]. As such, dilute colloidal systems can oft be described by the Einstein equation (Equation (1)), where viscosity increases linearly with the occupied volume fraction of colloid [38] (Figure 2A).

(1) $$\eta =\eta_{0}\left(1+\left[\eta \right]\phi \right)\;\mathrm{where}\;\phi =V_{h}C$$

where η is the viscosity of the solution (Pa·s), $\eta_{0}$ is the viscosity of the solvent (often water or saline) with no polymer (Pa·s), $\left[\eta \right]$ is the intrinsic viscosity of the colloid (2.5 for monodisperse hard spheres, -), and ϕ is the volume fraction of the solute (-). For polymers, volume fraction can be found from the product of $V_{h}$, the hydrodynamic volume of the polymer in solution (dL g^−1^) and C, the polymer concentration (g dL^−1^).

As the concentration increases, the colloids can begin to interact with each other. This leads to larger increases in viscosity, as particles can collide, attract and, in the case of polymers, entangle. These increased connections necessitate more complex descriptive models, which must now take into account the nature of the interactions, and thus the type of colloidal system. For particulate systems which do not overlap, the concept of excluded volume is extended, for example in the Krieger–Dougherty equation [39] (Equation (2), Figure 2A).

(2) $$\eta =\eta_{0}{\left(1-\frac{\phi }{\phi_{m}}\right)}^{-\left[\eta \right]\phi_{m}}$$

where $\phi_{m}$ is the maximum packing fraction; this value is 0.64 for monodisperse hard spheres [40]. This relatively simple description, however, becomes more complex for particles of differing shape, size and aspect ratio [41], deformable droplets with internal flow, as in an emulsion [42], and charged colloids [43].

For polymeric systems, as concentration increases the polymers not only interact, but overlap and entangle with each other [44]. This leads to larger, non-linear viscosity increases, and descriptive equations must again become increasingly complex. For still relatively dilute systems, the Huggins equation (Equation (3)) may be used to describe the viscosity [45] (Figure 2A).

(3) $$\eta =\eta_{0}\left(1+\left[\eta \right]C+K{\left(\left[\eta \right]C\right)}^{2}\right)$$

where K is the Huggins parameter (-). To describe more concentrated polymer solutions, the exponential Martin equation (Equation (4)) may become more appropriate [46].

(4) $$\eta =\eta_{0}\left(1+\left[\eta \right]Ce^{K\left[\eta \right]C}\right)$$

At high polymer concentrations, a power series expansion of Martin’s model, where each term describes an additional interpolymer interaction (Equation (5)), has been applied [47].

(5) $$\eta =\eta_{0}\left(1+C\left[\eta \right]\right)\left(1+KC\left[\eta \right]+\frac{{\left(KC\left[\eta \right]\right)}^{2}}{2!}+\frac{{\left(KC\left[\eta \right]\right)}^{3}}{3!}\ldots \right)$$

These equations all describe colloidal systems in a given state; however, as many of the interactions in colloidal systems are transient, they will change as the state of the system changes. In addition to temperature, one of the most important for biomaterials are the effects of applied forces, which often occur during application. For many colloidal systems, these applied forces, most often shear stresses, break the cohesive bonds, and align the colloids in the flow field [48,49,50]. This leads to a decrease in viscosity, and such materials are thus termed shear thinning. Descriptions of such systems include the Ostwald de Waele model (Equation (6)), describing the shear thinning region [51] (Figure 2B).

(6) $$\eta =k{\dot{\gamma }}^{n-1}$$

where $\dot{\gamma }$ is the shear rate (s^−1^), and k and n are constants; n<1 for a shear thinning fluid. The Cross model (Equation (7)) describes a larger region of shear rates [52] (Figure 2B).

(7) $$\eta =\eta_{\infty }+\frac{\eta_{0}-\eta_{\infty }}{1+{\left(k\dot{\gamma }\right)}^{n}}$$

where $\eta_{0}$ is the viscosity in the low shear region, where the applied forces are too low to disrupt the intercolloidal interactions (Pa·s), and $\eta_{\infty }$ is the viscosity in the high shear region, where all the bonds have been broken and the colloids are maximally aligned (Pa·s).

For flocculated particulate systems, where the degree of flocculation depends on the applied shear force, a broadening of the Krieger–Dougherty equation (Equation (2)) can be applied, which includes a shear rate term (Equation (8)) [43].

(8) $$\eta =\eta_{0}{\left(1-{\left[\frac{R_{0}\left(1+{\left(b\dot{\gamma }\right)}^{c}\right)}{a}\right]}^{3-D}.\frac{\phi }{\phi_{m}}\right)}^{-\left[\eta \right]\phi_{m}}$$

This description also takes into account the radius of the flocc, $R_{0}$ (m), and flocc density, D (-), which varies between 1 for very open, linear floccs to near 3 for dense floccs; a, b and c are system parameters.

Shear thinning occurs as a result of the imposed force breaking the interactions between the particles or polymers, however in some systems these interactions are such that a certain amount of force is needed to break them before the system flows. This property is known as a yield stress, and such materials behave as solids, or infinitely viscous, when the applied stress is that of the yield stress, then as fluids, often shear thinning, when above it [53,54]. This property may be described by the Herschel–Bulkley model (Equation (9)) [55] (Figure 2B).

(9) $$\left\{\begin{array}{c} \eta =\infty \;for\;\tau <\tau_{0}\;\\ \eta =\frac{\tau_{0}}{\dot{\gamma }}+k{\dot{\gamma }}^{n-1}\;for\;\tau >\tau_{0} \end{array}\right.$$

where τ is the applied stress (Pa) and $\tau_{0}$ is the yield stress of the system (Pa).

In all of the above descriptions, it is assumed that the system is at steady state, or reacts to the applied mechanical force instantaneously. However, many systems take a finite time to change their microstructures in response to application or removal or force [56,57]. Such materials are known as thixotropic, and the viscosity of these materials undergoing a step change in applied shear may be described by the stretched exponential model (Equation (10)) [43].

(10) $$\eta =\eta_{e,\infty }+\left(\eta_{e,\infty }-\eta_{e,0}\right)\left(1-e^{-\frac{t}{\lambda }}\right)$$

where $\eta_{e,\infty }$ is the eventual viscosity of the system if the shear was applied indefinitely (Pa·s), $\eta_{e,0}$ the initial viscosity just before the shear is applied (Pa·s), t is the time of shearing (s) and λ is a constant (s).

As concentrations increase further and interactions between colloids become stronger and more numerous, they can form three dimensional networks capable of storing energy, imparting elasticity to the system. Such systems, capable of both elastically storing and viscously dissipating energy, are known as viscoelastic [58]. The properties of these materials can no longer be described purely in terms of viscosity, itself the ratio of stress and shear strain rate, but also include strain magnitude and further time derivatives. The simplest models are the Maxwell (Equation (11)) and Kelvin–Voigt (Equation (12)) models.

(11) $$\frac{1}{E}\frac{d\sigma }{dt}+\frac{\sigma }{\eta }=\frac{d\varepsilon }{dt}$$

(12) $$\sigma =E\varepsilon +\eta \frac{d\varepsilon }{dt}$$

where σ is the stress (Pa) and ε is the strain (-), E is the modulus of elasticity (Pa) and η the viscosity (Pa·s). For many viscoelastic materials, particularly those with multiple interacting phases and components, these descriptions are too simplistic and are combined, for example in the Burgers model (Equation (13)).

(13) $$\sigma +\left(\frac{\eta_{1}}{E_{1}}+\frac{\eta_{2}}{E_{2}}\right)\frac{d\sigma }{dt}+\frac{\eta_{1}}{E_{1}}\frac{\eta_{2}}{E_{2}}\frac{d^{2}\sigma }{dt^{2}}=\left(\eta_{1}-\eta_{2}\right)\frac{d\varepsilon }{dt}+\frac{\eta_{1}}{E_{1}}\frac{\eta_{2}}{E_{2}}\left(E_{1}+E_{2}\right)\frac{d^{2}\varepsilon }{dt^{2}}$$

where the subscripts denote the properties of the different parts of the material.

Increased cohesion and structural complexity are integral to modern biomaterials, as their material and rheological behaviours can largely dictate their biomedical functionality. For example, shear thinning allows a material to be viscous *in situ*, while greatly reducing the forces required during application. Yield stresses and viscoelasticity are valuable properties designed into fluidic biomaterials, providing amongst other abilities an inherent capacity to enhance retention, site specificity and mechanical protection during application. Indeed, many popular structured fluid biomaterials, including hydrogels, microgel suspensions, creams and ointments, are highly viscoelastic. Thixotropy, while often undesirable, is an important property of many real microstructured systems. In addition to these properties, colloids and their assembled microstructures can provide further beneficial features, including increased lubrication and mucoadhesion. This increased structural complexity necessitates increased modelling complexity; unlike simple saline, descriptions of the flow of microstructured, colloidal biomaterials can no longer be described with a single number.

## 3. The Disruptive Forces in Spraying

Sprays are among the most intellectually challenging and practically important topics in fluid mechanics [59]. Spraying is encountered in a plethora of industries, including biomedicine (discussed in Section 1), agriculture [60,61,62,63,64], inkjet printing [65,66,67], spray painting [68,69,70], combustion engines [71,72,73] and firefighting [74,75], as well as various manufacturing methods, including spray drying [76,77,78], electrospraying [79,80,81], plasma spraying [82,83,84], thermal spraying [85,86,87] and spray coating [88,89,90]. Matching the diversity of applications are the multiple types of spray device, from large-scale, pressurised liquid ejected through a nozzle in agricultural spraying, atomisers for pulmonary drug delivery, air flows used in spray painting, to hand-operated metered-dose nasal spray devices. The ultimate aim of the process, and the method to achieve it, can also vary widely. In pulmonary nebulisers, small, easily inhaled drops are needed, but not so small that instantaneous evaporation occurs, with droplet size determining the targeted part of the airway [91,92]. Conversely in inkjet printing, the aim is to produce a fine jet and generally avoid the formation of small satellite droplets [67]. In agriculture, the goal is to achieve small, monodisperse droplets to evenly coat the desired vegetation, while avoiding fine drops that can be carried by the wind, a process known as spray drift [62]. Much research in this area involves changing the nozzle design, ejection pressure, and adding small amounts of additives to change the material properties [93,94]. Indeed, changing the flow rate through the ejection pressure has been found to be the most effective way of changing the droplet size distribution [92]. However, in many biomedical applications, particularly where materials are to be self-delivered by hand, these parameters may be far less flexible.

**Figure 3:**
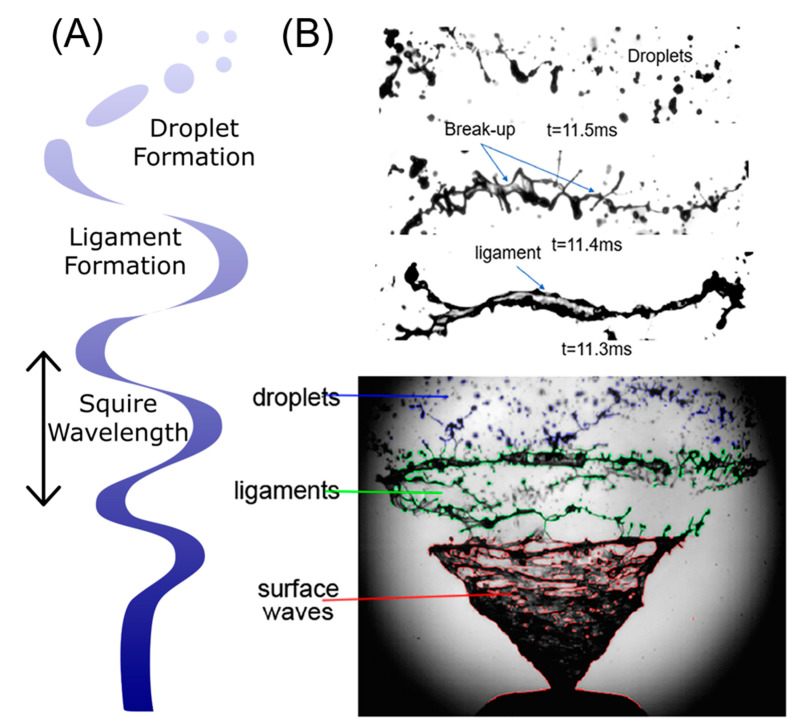
(**A**) Schematic outlining the successive breakup steps during spraying, (**B**) images of nasal spray application, highlighting the fluid morphology at each stage (adapted from [97]).

The process of spraying is, in essence, the formation of droplets from a bulk fluid. This occurs first by the formation of a jet or sheet from the bulk liquid, the breakup of this film into ligaments, and the further breakup of these ligaments into droplets (Figure 3) [95]. In the first step, the jet or sheet produced oscillates as a transverse wave through friction with the air, thicker at the peaks and troughs and thinner in the centre, characterised by the Squire wavelength [96]. This theoretical analysis, which agreed with experimental results for oil ejected through a conical nozzle, was based on independent variables both for the actuator, including initial film thickness and fluid velocity, and material properties, including fluid and interfacial tension [96]. Viscosity and other material properties are conspicuous by their absence. Following initial breakup, the sheet fragments contract to form ligaments, which then break up into droplets due to surface tension-driven Rayleigh instability [92].

A number of empirical equations have been developed for important spray parameters (Equation (14) [92], Equation (15) [98], Equation (16) [99] and Equation (17) [94]). These include expressions for average droplet size, with median droplet size, $D_{50}$, and Sauter mean diameter, SMD, being the most common, and spray angle, θ, which will also influence spray coverage.

(14) $$D_{50}=Cb{\left(\frac{\rho_{a}}{\rho_{f}}\right)}^{-\frac{1}{6}}We^{-\frac{1}{3}}$$

(15) $$SMD=CRe^{-0.183}We^{-0.442}{C_{d}}^{-0.422}{\left(\frac{\rho_{f}}{\rho_{a}}\right)}^{-0.05}$$

(16) $$tan\theta =C{\left(cos\left(90-\varphi \right)\right)}^{-1.39}Re^{1.11}{\left(\frac{d_{0}}{d_{p}}\right)}^{0.05}{\left(\frac{d_{0}}{D_{S}}\right)}^{0.32}{\left(\frac{d_{0}}{H_{S}}\right)}^{0.42}$$

(17) $$\theta =CRe^{0.39}$$

where C is a coefficient (-), b (-), φ (°), $d_{0}$ (m), $d_{p}$ (m), $D_{S}$ (m) and $H_{S}$ (m) are geometrical properties of the actuator, $\rho_{a}$ and $\rho_{f}$ are the densities of air and the fluid (kg m^−3^), respectively, We is a Weber number (-), Re is a Reynolds number (-), and $C_{d}$ is a discharge coefficient (-); a function of Re.

Notably, the material properties considered in these expressions are the fluid density, surface tension (through the Weber number) and viscosity (through the Reynolds number). Interestingly, Equation (14) has no viscous term at all; however, this study only considered Newtonian water-glycerol mixtures, with viscosity ranging between 0.001 and 0.032 Pa·s [92]; many modern colloidal biomaterials, including polymeric and ceramic fluids, have viscosities many orders of magnitude higher [100]. Equations (15) and (17) are from studies on Newtonian oils with a single viscosity value [94,98]. Equation (16) is based on relatively dilute solutions of sodium carboxymethylcellulose, a hydrophilic polymer. These colloidal systems were found to be shear thinning, and were characterised using the Ostwald de Waele equation (Equation (6)). The Reynolds number in Equation (16) is thus a generalised version in terms of k and n. It may be possible to extend this idea for other non-Newtonian fluid models, for example a generalised Reynolds number can be defined for fluids described by the Herschel–Bulkley model (Equation (9)) [101]. However, for structured fluids which display significant thixotropy and/or viscoelasticity, and thus cannot be described purely by relations between shear stress and shear rate, simply replacing the Reynolds number with a generalised version may be insufficient.

**Figure 4:**
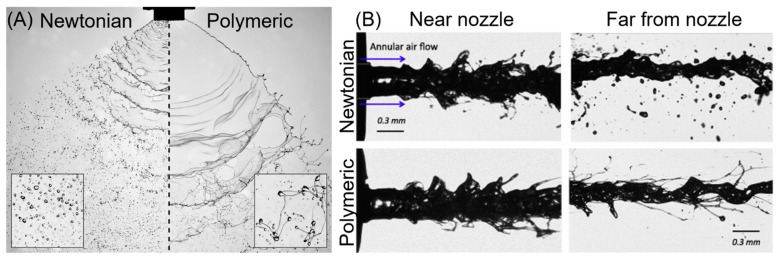
Spray geometry of Newtonian fluids and dilute polymer solutions from (**A**) a flat fan nozzle (adapted from [106]) and (**B**) a coaxial air flow nozzle (adapted from [111], reproduced with permission from Elsevier).

The effects of viscoelasticity on spraying have not gone unnoticed; indeed, for decades small amounts of polymer have been added to fuel to prevent misting [102,103], and to agricultural sprays to minimise fine droplets and limit spray drift [104,105]. It has been reported that the addition of polymers both increases the average droplet size and broadens the droplet size distribution [106,107,108]. The viscoelasticity imparted by the polymers inhibits droplet formation by stabilising the formed filaments, resulting in an increase in average droplet size and, critical for the afore mentioned applications, suppressing fine droplet formation [63]. Filament stabilisation also leads to a different morphology; instead of droplets, many threads, ropes and webs are formed [109,110], an effect that appears to be independent of spray nozzle geometry (Figure 4).

While not yet comprehensive, some work has related the physicochemical properties of colloids in dilute solution with spray parameters, mainly for polymers as drift control adjuvants. An increase in molecular mass greatly increases the extensional viscosity [63], the parameter most strongly implicated in the change in morphology and characteristics of the spray [93,109]. Increased molecular mass also raises the shear viscosity and lengthens to time taken for the polymer to change conformation [111], leading to an increase in average droplet size [112]. The flexibility of the polymer chain is also influential, as more flexible polymers increase the extensional viscosity far more than rigid ones at the high extension rates seen during the spray process [113]. As such, the polymer concentration required to completely collapse the spray cone into a jet is lower for flexible polymers. Charged polymers seem to be more resilient to mechanical degradation, however the effect on the spray parameters themselves appears negligible, though more systematic studies that alter polymer charge only while keeping all other parameters constant is needed [114,115]. Polymers that incorporate hydrophobic groups into their chains can greatly increase the extensional viscosity, but only above a the critical concentration where inter-polymer interactions dominate over intra-polymer attraction [116,117].

It is important to note that in these studies, small amounts of polymer have been added to materials that already sprayed well, often to limit production of fine droplets. However, increasing polymer molecular weight and concentration, often required to achieve the required properties of biomaterials, will have a deleterious effect on the spray, leading to spray sheet collapse and formation of a jet [23,64,113]. Similar results may be expected for other concentrated colloidal systems. The challenge thus remains of whether highly viscoelastic biomaterials, which do not inherently spray well, can be made to do so without compromising the material properties that enable their biomedical function.

## 4. Towards Spraying Structured Fluid Biomaterials

Both structured fluids and spraying have unique advantages for controlled delivery, and combining them has the potential to provide new therapeutic options for a vast range of diseases. Spraying can be used to deliver treatments directly to specific areas, for example to the skin, nose, mouth, anus and vagina, as well as to the lungs and gastrointestinal tract. Additionally, structured fluids can be sprayed onto these surfaces to act as depots for mucosal absorption, for systemic application [118,119] or near-site delivery, as in intranasal delivery to the brain [120,121]. These materials can be used alone as therapeutic delivery vehicles, using the designed microstructure to control release, or utilising the response of many colloidal systems to stimuli such as light, temperature, pH and enzymes. Additionally, these material properties of structured fluids may be combined synergistically with cutting edge delivery techniques, including micro- and nano-sized particles, fibres, tubes, capsules, dendrimers, lipids, micelles and liposomes [122,123]. Further, this approach is highly compatible with emerging technologies, including stem cell [124], DNA [125] and RNA therapies [126], providing potential to impact the greatest medical challenges of our time, including cancer [127], chronic wounds [128] and infections [129].

However, creating a readily sprayable material with all of these desired functional properties is not trivial. Most current biomedical sprays are simple inviscid fluids; however, many of the beneficial drug and cell delivery properties of colloidal biomaterials arise from their cohesive forces, creating structured, entangled and cross-linked networks. Conversely, spraying is a disruptive process, breaking a bulk fluid into individual droplets. Given these conflicting aims, is it possible to achieve both simultaneously; can we structure biomaterials to spray well whilst maintaining functionality?

As discussed in Section 3, current correlations for spray metrics revolve around a Reynolds and/or Weber number, implicating the viscosity and surface tension of the material, respectively, as key material properties. However, these dimensionless numbers are in fact the ratios of these forces and the inertial forces acting on the system, suggesting that increasing the ejection speed of the fluid will enhance sprayability. Indeed, increasing the pressure head has been, and may continue to be, a simple and viable route to maintain sprayability in large scale industries such as agriculture. However, many biomaterial sprays are designed to be self-administered, or administered by a carer or clinician, by hand. Even in machine-based sprays, such as pulmonary nebulisers, it may be technically impossible, or medically inadvisable, to greatly increase the fluid velocity. Another consideration is that applying excessive mechanical forces to colloidal systems may permanently disrupt the created microstructure, eliminating the useful rheological properties, as well as destroy any loaded cells or delicate therapeutic agents. For all structured systems then, but most especially those for biomedical applications, it may thus be most appropriate to engineer the material itself to spray well.

While excessive force is to be avoided, the spray process is axiomatically disruptive, and thus the way the material responds to these forces will be crucial in order to both spray well and be functional. Key considerations here will be the types of cohesive interactions being broken during spraying, whether they recover, to what extent, and how quickly. For example, breaking most covalent bonds through the spray process is likely to result in permanent loss of material properties. This is seen during agricultural spraying of dilute polymer solutions in a process known as mechanical degradation, leading to rapid loss of performance [130]; again highlighting the need to avoid excessive spray forces. The interactions broken should instead be reversible; for example, Van der Waals forces, hydrogen bonds, ionic attractions, metal coordination, hydrophobic interactions and steric entanglements are colloidal forces which may both be broken with modest force, then reform when the force has been removed [131]. This process is often referred to as self-healing, and is an important property of many biomaterials, including colloidal systems for injection and bioprinting [131,132,133,134].

As discussed in Section 2, many biomaterials are thixotropic; that is, they take a finite time to rearrange their microstructures in response to a change in applied force. This will be important in sprayed biomaterials both during and after application. During spraying, not only will the applied force need to be sufficient to break up the material into droplets, but this microstructural rearrangement will have to take place quickly, relative to the time over which the spray process occurs. In a recent study, attempting to extend Equation (14) to dilute polymer solutions, a colloidal system, Gaillard et al. [106] proposed Equation (18).

(18) $$D_{50}=C_{1}b{\left(\frac{\rho_{a}}{\rho_{f}}\right)}^{-\frac{1}{6}}\left(We^{-\frac{1}{3}}+C_{2}DeWe^{k}\right)$$

where De is a Deborah number, the ratio of the relaxation time of the system, a measure of how quickly the microstructure of the material changes in response to a change in applied force, and the time over which the process takes place. A low Deborah number, De≪1, may thus be an important factor to consider when designing structured fluid biomaterial sprays.

Once *in situ*, it will be important that the biomaterial fully recover its microstructure, and thus material properties, in a timely manner. This timescale may depend on the application, but for many of the applications discussed in Section 1, including spraying biomaterials onto the skin, into the eye or the internal mucosa, the biomaterial should fully recover in the timescale of ms–s, to prevent any flow, dripping, and loss of coverage.

As discussed in Section 2, many of the interactions that give rise to the useful properties of structured fluid biomaterials occur at the molecular scale, either between the colloid and solvent, or between colloids. However, the maximum level of breakup during spraying needs to be on the order of the size of the final droplets, μm–mm. This may present an opportunity to engineer structured fluids such that they possess the short-range interactions required to give rise to useful biomaterial properties, but limit the medium- to long-range order which may inhibit spraying.

## 5. Conclusions and Perspective

Utilising increasingly structured fluids is becoming ever more popular for controlled delivery, giving higher residence times, site specificity and ability to tailor release. Equally, spray delivery is emerging as a powerful application method, able to apply a thin layer of material over hard-to-reach anatomical locations with complicated surface geometries. Combining these approaches has the potential to lead to great advances in the delivery of drugs and cells, particularly to the nose, mouth and skin. However, highly viscoelastic biomaterials pose a unique challenge, as the cohesive forces which produce the required structure oppose the disruptive forces of spraying. Unlike many industries, increasing the spray pressure may be inadvisable for biomedical applications, and tweaking nozzle geometries is unlikely to have a great effect. Engineering the microstructure of these structured fluids may thus be the most effective means to increase both sprayability and functionality. However, concepts beyond viscosity and surface tension will be needed to account for the significantly non-Newtonian behaviour arising from the complex microstructures of colloidal biomaterials. The scales of both length and time will need to be carefully considered in order to make significant advancement in structured fluid spraying, an endeavour which will benefit not only biomaterials, but all spray industries. Though unlikely to be straight forward, by considering all of these factors to conceive of new design principles, the authors firmly believe we can structure biomaterials to both spray well and maintain functionality.

## Data Availability

Not applicable.
